# Evaluation of the Hygienic Quality of the Gastronomic Offer of a Coastal Tourist Destination: A Study in San Pablo, Ecuador

**DOI:** 10.3390/foods11060813

**Published:** 2022-03-12

**Authors:** Verónica Guadalupe-Moyano, César Villagómez-Buele, Mauricio Carvache-Franco, Wilmer Carvache-Franco, Tito Ramón-Casal

**Affiliations:** 1 Facultad de Ingeniería Química, Universidad de Guayaquil, Guayaquil 090514, Ecuador; veronica.guadalupem@ug.edu.ec (V.G.-M.); cesar.villagomezb@ug.edu.ec (C.V.-B.); tito.ramonc@ug.edu.ec (T.R.-C.); 2 Facultad de Turismo y Hotelería, Universidad Espíritu Santo, Samborondón 092301, Ecuador; mauricio2714@hotmail.com; 3 Facultad de Ciencias Sociales y Humanísticas, Escuela Superior Politécnica del Litoral, ESPOL, Guayaquil 090903, Ecuador

**Keywords:** San Pablo, gastronomy, hygienic quality, food offered, good manufacturing practices

## Abstract

In Ecuador, the San Pablo commune is one of the main tourist destinations in the Ecuadorian coastal region, recognized for its scenic landscapes and its gastronomy based on fish and seafood. The objective of this study is to evaluate the hygienic quality of the food offered in this location. Hence, five local restaurants were audited for compliance to good manufacturing practices (GMP), considering requirements for personnel, raw materials, facilities, equipment and utensils, quality assurance and storage conditions. Concurrently, four groups of ready-to-serve foods were sampled: rice, fish, natural juice, and raw salads to analyze total coliforms, *Staphylococcus aureus*, *Escherichia coli*, and *Salmonella* spp. The results confirmed the absence of *Staphylococcus aureus*, *Escherichia coli*, and *Salmonella* spp. However, values outside the norm for total coliforms were quantified in three food groups. There was an average compliance of 66.46 ± 16.67% regarding GMP with no significant difference in compliance between the six groups of requirements. These results indicate that work is needed to improve GMP, increase the hygienic quality of food and enhance the gastronomy offered in San Pablo.

## 1. Introduction

The current trend in travelers’ activities from all over the world favors the consumption of local foods. In fact, from a tourist perspective, food structures the tourist’s day since a large part of it is devoted to deciding where and what food and beverages to consume [1].

According to Brokaj [1], the motivations for consuming local foods include five factors: taste quality, authentic experience, rural development, concern for health, and learning. In addition, in developing gastronomy as a tourist attraction, consumers’ quality includes food and service [2]. In this regard, in coastal destinations, the main motivations of tourists are resting and enjoying the beach, the sun, and the typical gastronomy of the area [3].

The link between tourism and the development of gastronomy has led to new opportunities for many local food products [4]. For this reason, Zhang et al. [5] affirmed that the sustainability of rural progress has been increasingly linked to local food, which plays an indispensable role in the preservation of traditional culture and increase in tourism [6,7]. Moreover, the environment plays an indispensable role in the local gastronomic tourism package as visitors also appreciate beautiful landscapes and high biodiversity in the region [8].

Regarding the high quality and hygiene of food, its remarkable importance on the image of a destination, tourist flow, country’s income, foreign direct investment, and visitors’ health must be considered [9]. Studies show that, over the years, many foodborne illnesses have been reported due to the consumption of contaminated non-homemade food, mainly due to low hygiene standards. Thus, preventive actions are recommended to maintain standard hygienic procedures for preparing, cooking, and handling food [10].

In reference to the community studied in this research, San Pablo, Ecuador, is a coastal locality with high fishing activity and is one of the main tourist destinations on the country. This tourist destination, known for its beautiful landscapes, offers a gastronomy rich in fish and seafood dishes, bringing an enjoyable place to be visited for national and international tourists. Nevertheless, it has to be considered that developing countries have needs not yet covered in essential services and infrastructure [11], which constitutes a significant deficit for the tourist and gastronomic appeal of a locality such as San Pablo, Ecuador.

In this context, considering food hygiene as a critical issue in the construction of quality for the gastronomic offer, up to the present time, very few studies have been carried out that address an analysis of the quality and hygiene of typical foods of a coastal region, specifically in Ecuador. Therefore, to attend this research gap, the objective of this study is to measure the level of quality of the gastronomic offer of a coastal destination. Two main aspects are considered in this research: compliance with good manufacturing practices (GMP) and the microbiological quality of the foods offered.

## 2. Literature Review

### 2.1. Gastronomy and Culture

Throughout the last decade, tourism has become an important element for improving various aspects in a country, taking advantage of gastronomy as a recursive element, since food functions as a metaphor for the construction and expression of ethnicity and cultural identity [12]. Along these lines, Ellis et al. [12] indicate that food is often used for rural development and, therefore, execute a remarkable role as a main interest in tourism. In the process of consuming and experiencing locally sourced or traditionally grown products/ingredients, visitors embrace the values associated with local identity codes and satisfy most of their expectations when it comes to cultural experiences [13,14].

These days, traditional establishments monopolize the importance of restoration as a source of heritage and culture, but even luxury restaurants have the opportunity to promote products of the terroir and rural gastronomic tourism, the gastronomization of rural destinations and the increase in the media capital of the destination [15]. On this subject, Timothy [16] affirmed that heritage tourism would continue to develop, and an increasing number of destinations will realize the socioeconomic potential of sharing their folklore with visitors.

### 2.2. Tourist Hygiene Expectations

The increase in gastronomic demand has impacted the average tourist’s point of view and expectations in certain aspects, such as the quality of food and hygiene. This consideration is particularly important for the gastronomic tourist, since they plan trips, stay longer, and spend more discretionary money when traveling [17]. According to Almohaimmeed [18], hygiene quality significantly impacts customer satisfaction, supporting that foodservice hygiene is one of the three primary considerations when consumers choose a place to have dinner [19].

Increasing consumer awareness and knowledge of the quality and safety of street food can counteract inappropriate hygiene practices in different establishments [20]. This factor is vital in restaurants operated by ethnic groups, with significantly higher rates of inspection and critical violations due to practices that may involve techniques or methods that are not validated by local regulatory systems [21]. Therefore, according to Impraim et al. [22], regular education and professional training have a significant impact on food safety and hygienic practices of catering service providers and restaurant managers in the industry of tourism.

### 2.3. Ecuadorian Regulatory Entities of Food Quality

In Ecuador, the rules and regulations are administered by a national entity called the Ecuadorian Standardization Service (INEN), which seeks to preserve the safety and health of people, animals, and plants, as well as the environment and national security [23]. With the support of INEN, the National Agency for Food Regulation, Control and Surveillance (ARCSA) acts as the main auditing entity in the country to ensure the application of a series of norms and standard procedures, with the aim of preserving the excellent quality and safety of food for human consumption [24]. Subsequently, and as a specialized agency in direct control of catering establishments, we find the Ministry of Tourism of Ecuador, which seeks to support the tourism promotion of various locations, as well as preserve certain quality standards in various related areas [25].

### 2.4. Sanitary Quality in Restaurants

Regarding quality in food tourism, the handling of ingredients and processed dishes includes a wide variety of safety measures that prevent inconveniences such as food poisoning and hygiene depreciation around the world [26].

According to ARCSA [24], all food received in a cafeteria or restaurant must be adequately protected and handled to avoid unintentional adulterations. To do this, it establishes control points and precautionary measures that are focused according to the type of food, optimal temperatures, and storage systems. Thus, an effective and credible food safety regulatory system remains a critically important role in public policy [27]. In catering establishments, it is essential to maintain a well-prepared and trained staff with good manufacturing practices (GMP) [28]. Because of this, non-compliance with food control and hygiene protocols, as well as HACCP in gastronomy tourism, can contribute to disease outbreaks [29].

In recent years, people have become more concerned about food safety and sanitary hygiene [30]. Additionally, as many authors have discussed, the importance of hygiene and the contribution of safety measures in food processing is too relevant for gastronomic tourism to avoid. However, it is also recommended to take into account that it is difficult to control the process when methodologies that concern the degree of hygiene are followed, which, in some cases, could result in changes to traditional recipes that could lead to significant cultural heritage losses [31]. Heo and Bae [32] state that a hygiene grade certification system can help customers to track the sanitary quality and help further control food establishments. In this case, the government should provide the relevant policy for certified restaurants and relevant education and promotion to customers.

### 2.5. Standards for the Implementation and Monitoring of GMP

The International Organization for Standardization (ISO) is a worldwide federation of national standards bodies (ISO member bodies) [33]. A hybrid of the ISO 9001 Quality Management System and Hazard Analysis and Critical Control Points (HACCP), ISO 22000 has been developed as an international solution to improve food safety in the food industry. ISO 22000, also known as the Food Safety Management System (FSMS), is an international auditable standard [34]. The technical standard ISO/TS 22002-2 delves into the prerequisites for food safety during food preparation (the GMP for catering). It standardizes the correct procedure of various points pertinent to food handling and processing and their work areas to preserve food safety in restoration food establishments [35]. Some of these regulations are work areas and buildings; distribution of facilities and equipment; human circulation flows, internal structures and accessories; food storage, packaging, and packaging materials; chemicals not used in food; condition and quality of basic services (water, air, energy); trash deposit; personal hygiene and sanitation of equipment/facilities; sanitation equipment; management of purchased materials; cross-contamination prevention measures; and pest control.

In addition, ARCSA addresses most of the aspects mentioned earlier regarding the food safety and hygiene of preparations to evaluate restaurants and cafes as a regulatory system in Ecuador, including GMP, health, and legal issues, through the norm ARCSA-DE-067-2015-GGG, part III and the External Instructions for the Evaluation of Restaurants/Cafeterias IE-E.2.2-42 [24].

### 2.6. Foodborne Illnesses

Currently, foodborne diseases continue to be a significant threat to public health, as they are transmitted through the consumption of contaminated food, which proliferates pathogens among consumers [36]. These diseases are infectious or toxic and are caused by bacteria, viruses, parasites, or chemicals (Table 1), which enter the body through contaminated food or water [37]. According to the World Health Organization (WHO), they constitute one of the most widespread health problems globally and are a fundamental cause of reduced productivity and absenteeism from work [37]. Recent studies by Lee and Yoon [38] have shown that norovirus causes the highest number of foodborne illness cases worldwide, followed by *Campylobacter*, *Salmonella*, and *Listeria monocytogenes*.

The concept of contamination is understood as any matter incorporated into food without being part of it that can produce diseases in those who consume it [24]. The Food and Drug Administration (FDA) report of recalls, conducted by food processors to examine contamination or potential contamination of food, shows a total of 1098 recalls, from January 2017 to June 2021, where 42% correspond mainly to *L. monocytogenes*, *Salmonella*, *C. botulinum*, *E. coli* and other causes such as norovirus, *Cyclospora*, histamine, mycotoxins, foreign matter, and heavy metals [39]. The origins of microbial contamination are diverse, for example, soil, air, equipment surfaces, packaging material, and personnel [40]. Moreover, pollutants can be biological, chemical, and physical [24]. In this way, chemical contamination of food is a serious concern due to the serious health risks involved [41]. According to Nerin et al. [42], food contamination can result from different situations during any food process, from contaminants already existing in raw materials to packaging processes.

### 2.7. Importance of Microbiological Evaluation

The implementation of microbiological criteria focuses on providing tools for the management of quality systems, enabling the discrimination of defective batches, and controlling industrial aspects [43].

Regarding food sampling for microbiological analysis, Van Schothorst et al. [44] stated that sampling plans could be designed to evaluate a microbiological criterion. A microbiological assessment scheme can provide insight into microbiological contamination in the production process and to identify faults in the food safety management system [45]. This information supports the assertion of Buchanan [46], who indicated that the microbiological data acquisition and archiving routines are carried out to compile microbiological profiles of food and the virulence of foodborne pathogens, to develop strategies and criteria to ensure microbiological safety. On the other hand, Valero et al. [47] identified environmental sampling as an effective procedure to verify the correct implementation of food safety control systems in catering establishments.

### 2.8. Hygiene Indicator Microorganisms

According to Ray and Bhunia [48], the concept of indicator bacteria was introduced to measure the sanitary quality of food, and its purpose was to indicate the possible presence of enteric pathogens (for example, *Salmonella*). Subsequently, the indicator microorganisms determine whether the food has been exposed to risky contaminating conditions with a pathogen or kept in conditions that would allow the proliferation of pathogens [49]. Thus, Camargo et al. [50] concluded that microbiological tests are an essential quality management tool in the food industry. Some common indicator microorganisms are *Enterobacteriaceae*, *Listeria* spp., aerobic plate count, coagulase-positive staphylococci and coliforms, *Escherichia coli*, which, according to various authors, in high concentration counts in food, raise concerns about the possible presence of food-borne outbreaks [51,52].

Indicator microorganisms are helpful to control the hygienic quality of food and sources of water for human consumption [53]. Findings by who et al. [52] show a high probability of the transmission of pathogenic bacteria from the food handlers to customers during food preparation and service. When hygiene programs involving indicator microorganisms are implemented, tests before and after critical control points of food processing are crucial to determining quality [54]. Moreover, Jay [55] stated that coliforms are used as indicators for dry foods and pasteurized ingredients, while *E. coli.* is generally applied to fish, meat, frozen, cooked, or precooked foods, and water.

## 3. Study Area

San Pablo is a coastal destination in Ecuador of the International “Route of the Sun” also known as the “Route of the Spondylus” [56]. Its climate is tropical and warm with an annual average temperature of 24.4 degrees Celsius because it is located in the coastal region on the shores of the Pacific Ocean, and it does not rain much during the year. Humpback whales can be observed on the coasts of San Pablo in the summer. Along the beach, there are different birds such as the white heron, present during the fishermen’s work, and butterflies.

The gastronomy of San Pablo is based on fish and seafood. The desirable dishes are fish casserole, seafood rice, breaded, popped, or fried shrimp, breaded fish, and ceviche [56].

## 4. Materials and Methods

### 4.1. Microbiological Measurement

For this study, four food groups widely eaten in the commune were considered: cooked rice, fresh fruit juice, fresh salad, and cooked fish. They were selected from five restaurants in the main tourist area of San Pablo. Two samples of 60 g were taken from each food group and restaurant (ten samples from each food group and a total of forty samples were taken) placed in sterile plastic bags for solid samples and sterile plastic containers for liquid samples, unified and homogenized to make a composite sample for each food group, resulting in a total of four composite samples for microbiological analysis. Cold containers were used to ensure storage until the samples were delivered to the laboratory. AOAC and BAM methods were followed to determine the total coliforms (AOAC 21st 991.14 ME04- PG20- PO02-7.2 M), *Staphylococcus aureus* (AOAC 21st 2003: 07), *Escherichia coli* (BAM 8th (ME08-PG20-PO02-7.2 M), and *Salmonella* spp. (AOAC 21st 967.26 (ME20-PG20-PO02-7.2 M). The analyzes were carried out in duplicate.

### 4.2. Good Manufacturing Practice Audit

Five out of ninety restaurants in the municipality of San Pablo were audited for compliance with the GMP. One-hundred-and-thirty-five points of the Ecuadorian law that regulate GMP were verified in situ (ARCSA-DE-067-2015-GGG, part III and the External Instructions for the Evaluation of Restaurants/Cafeterias IE-E.2.2-42). Table 2 shows a summary of the items required by Ecuadorian law. The level of compliance was tabulated as the percentage of items completed versus the total audited. Following the qualification criteria of the External Instructions for the Evaluation of Restaurants/Cafeterias IE-E.2.2-42, if a restaurant achieves 90 to 100% compliance with the GMP, it will be considered as level A, from 80–89.9% as B, if it reaches 60 to 79.9% as C, and it is less than 60%, it will be considered insufficient.

### 4.3. Statistical Criteria

The Military Standard Table (MIL-STD-105D) was the statistic tool for selecting the number of restaurants to be audited and for taking samples for microbiological analysis. In this case, the population is 90 (the total number of restaurants in San Pablo, Ecuador). A lot size from 51 to 90 was considered and the special level of inspection was S-3, in which the letter C was chosen corresponding to the sample size of 5 restaurants.

The analysis of variance (ANOVA) test was used to determine whether there was a significant difference between the results of requirement groups of the GMP standard with 95% confidence (*p* < 0.05). To verify homogeneity of the means, the multi-range test was used, whose method used is Fisher’s minimum significant difference (LSD).

For statistical analysis, Statgraphics Centurion XVI software (StatPoint Technologies, Inc., Warrenton, VA, USA) was used.

Regarding microbiological data, although the number of samples taken are representative, making a composed sample per food group leaves few samples for microbiological analysis. For this reason, it was not possible to carry out a statistical analysis of them. The results shown should be considered as an indicator of the response to the level of GMP compliance. Deeper microbiological analyses should be performed for greater statistical management.

## 5. Results

### 5.1. Microbiological Analysis

The microbiological results are shown in Table 3. Results reveal coliforms in three of the four food groups evaluated in quantities that exceed the national regulation. All the other microorganisms evaluated in the food samples were within the norm.

The results of total coliforms coincide with those obtained by Salazar-Llorente et al. [57], who also evidenced the presence of this microbial group in foods popularly consumed in the main cities of Ecuador. Additionally, they coincide with those of Orden-Mejía et al. [58] for a sampling carried out in shell ceviche in the city of Guayaquil-Ecuador. However, the results corresponding to *S. aureus*, *E. coli*, and *Salmonella* spp. do not coincide with the other authors since all these results were within the values required by the legislation. The presence of total coliforms reveals that the manufacturing practices in the sampled restaurants could have flaws and that the food is susceptible to contamination with other pathogenic bacteria since this microbial group is considered an indicator of hygiene [49]. Compliance with the reference standard for the other specific bacteria analyzed does not mean that the food, in general, is safe since the results are a sampling product. As the total coliforms exist outside the norm in various food groups, it is suggested to carry out a more extensive study.

Results such as these indicate an urgent need to improve the current handling practices that ensure food safety.

### 5.2. Good Manufacturing Practice Audit Results

**Figure 1:**
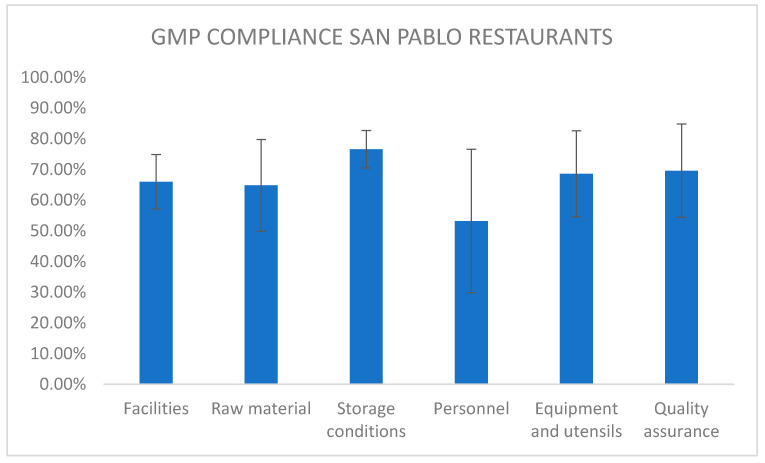
GMP compliance of San Pablo restaurants.

The GMP audit carried out on five randomly chosen restaurants in the study area reveals partial compliance with the requirements of the Ecuadorian standard, which have been grouped into equipment and utensils, personnel, storage, raw materials, facilities, and quality assurance (Figure 1). The greatest need for improvement is evidenced in the requirements relating to personnel with 53.2% compliance (education, health status, staff behavior, hygiene, and protection), although the standard deviation is very large (±26.1%) because the level of compliance is very variable from one restaurant to another; aberrant values were not eliminated to have a complete idea of the level of real variability. Requirements of personnel were followed by raw materials with 64.80 ± 16.7% compliance, facilities with 66 ± 9.9%, equipment and utensils with 68.6 ± 15.7%, quality assurance with 69.6 ± 17.0%, and storage conditions with 76.6 ± 6.9%, giving a general compliance of 66.46% ± 16.67, with no significant difference in the fulfillment of each group of requirements (*p* < 0.05). The high standard deviations indicate that the implementation of GMP is possible in just some the total restaurants; nevertheless, it implies that the majority of restaurants need to acquire more knowledge for their people in this subject and/or need a budget intended for improvement. It is there that the local authorities could intervene and support to achieve enhancement.

The partial fulfillment of the evaluated points could explain the presence of total coliforms in the analyzed samples and warn about a possible susceptibility to contamination with pathogenic microorganisms through cross-contamination. Hence, it is necessary to work in all the areas evaluated to improve practices, especially in the requirements regarding personnel, since it is the one with the lowest percentage of compliance. With the values achieved, four of the five audited restaurants are ranked in category C, and one in category B, according to the scale of the External Instructions for the Evaluation of Restaurants/Cafeterias IE-E.2.2-42 of the ARCSA, which indicates a need for improvement in good manufacturing practices in the restaurant sector of the municipality of San Pablo.

## 6. Conclusions

The gastronomy of San Pablo is well appreciated by consumers who seek its rich offer of preparations based on seafood. However, there is evidence of total coliforms in three of four food groups analyzed, which indicates an insufficient level of hygiene that must be corrected to avoid possible contamination with specific pathogenic bacteria.

In the present study, the presence of *Staphylococcus aureus*, *Escherichia coli*, and *Salmonella* spp. was not evidenced in the food samples analyzed, which is a good precedent to generate consumer confidence. However, when the total coliforms exceed regulatory standards, since this microbial group is an indicator of hygiene, there is a susceptibility to the contamination of food during the same preparation process with pathogenic microorganisms.

Furthermore, it is necessary to carry out more in-depth and broader microbiological studies of the gastronomic offer of San Pablo that includes the analysis of food, living and inert environments, and surfaces to detect the primary sources of contamination and prioritize their improvement.

The audit results can guide us on the requirements of good manufacturing practices that must be reinforced in San Pablo restaurants, some of which require investment and others which do not. The training of food-handling personnel will reduce the chances of cross-contamination and the implementation of control measures with affordable costs. In general, the results indicate the need to improve the manufacturing practices of the six groups of requirements.

The high standard deviation indicates that the implementation of GMP varies from one restaurant to another. The low compliance of four of the five restaurants could be based on a lack of knowledge or budget. It is there that the local authorities could intervene and support to achieve improvement. The present study wishes to promote an improvement in environmental and handling conditions to ensure food safety and promote gastronomic tourism in the town.

The theoretical contribution that this study presents to the academic literature is the result of the hygienic evaluation of the gastronomic offer of a coastal destination such as San Pablo based on the GMP compliance assessment. However, the element of microbiological sampling, similar to the contribution of Orden-Mejía et al. [58], is added. The results of both variables could be related, taking for granted that there is the presence of total coliforms in food, a microbial group considered an indicator of a hygiene level of low compliance to GMP.

The main limitation of the present study was a reduced microbiological food sampling. Hence, there was insufficient data to allow statistical correlation analyzes between the presence of pathogenic microorganisms and the results of good manufacturing practices in food-processing areas. The conclusions presented are based on the sampling results obtained, and they are related to GMP from a conceptual criterion of safety, hygiene, and cross-contamination, rather than on statistical validation.

Therefore, we were able to verify that a breach of GMP is generally present across the six groups of requirements with no significant difference between them, and it is a future line of research to address the great standard deviation in GMP compliance between restaurants. Additionally, the statistical correlation between microbiological data and GMP compliance must be determined.

## Figures and Tables

**Table 1:** Leading causes of foodborne illness worldwide.

Bacteria	*Campylobacter* spp.	One of the main foodborne pathogens, with serious or fatal effects. Symptoms: fever, headache, nausea, vomiting, abdominal pain, and diarrhea. Main contamination sources: raw milk, raw or undercooked poultry, and drinking water.
	*Escherichia coli* enterohemorrhagic	Common foodborne pathogen, with serious or fatal effects. Symptoms: fever, headache, nausea, vomiting, abdominal pain, and diarrhea. Main contamination sources: unpasteurized milk, undercooked meat, and fresh fruit and vegetables.
	*Listeria* spp.	Infrequent pathogen that can infect anyone with mild symptoms, except for pregnant women, infants, children, the elderly, and people with weakened immune systems, for whom is proven to become a dangerous infection. It can develop in refrigeration conditions. Symptoms: headache, stiff neck, confusion, loss of balance, and seizures, as well as fever and muscle aches. Main contamination sources: unpasteurized dairy products and various prepared foods.
	*Salmonella* spp.	Most known foodborne pathogen, with serious or fatal effects. Symptoms: fever, headache, nausea, vomiting, abdominal pain, and diarrhea. Main contamination sources: eggs, poultry, and other animal products.
	*Staphylococcus aureus*	Most dangerous bacteria among all staphylococci. It causes skin and bone infections, endocarditis, pneumonia, toxic shock syndrome, and food poisoning. Symptoms: vary from the type of infection. In the specific case of food poisoning, it usually causes nausea and vomiting, diarrhea, and fever. If the person loses too much fluid, dehydration may show up. Main contamination sources: the bacteria can enter the body through wounds or cuts on the skin, nose, and food. It is commonly passed through contact with different surfaces or contaminated foods.
	*Vibrio cholerae*	Cause cholera in humans, which is transmitted by ingestion of contaminated food or water. Symptoms: abdominal pain, vomiting, and profuse watery diarrhea, which can lead to severe dehydration and death. Main contamination sources: rice, vegetables, millet porridge and various kinds of seafood.
Virus	*Hepatitis A*	Causes a persistent liver disease that weakens its function in the long therm. Symptoms: fatigue, nausea, abdominal pain, clay-colored bowel movements, loss of appetite, low-grade fever, dark-colored urine, joint pain, yellowing of the skin and jaundice, severe itching. Main contamination sources: it is usually transmitted through contaminated food (by manipulation of an infected individual), ingestion of fresh vegetables and/or fruits, raw or undercooked shellfish, among other un products.
	*Norovirus*	Causes an infection called gastroenteritis, which inflames the stomach and intestines. Symptoms: nausea, explosive vomiting, watery diarrhea, and abdominal pain. Main contamination sources: similar to *Hepatitis A*, is transmitted through consumption of different undercooked or mishandled foods.
Parasites	Flukes	Found in fishery products, and an example of a common parasite that transmits solely by food consumption.
	*Echinococcus* spp. and *Taenia solium*	Transmitted through ingredients, food, or direct contact with animals.
	*Ascaris, Cryptosporidium, Entamoeba histolytica,* and *Giardia*	These parasites are introduced in the food chain through water or soil and can contaminate fresh produce.
Chemical substances	Heavy metals	Cause neurological and kidney damage. Some examples are lead, cadmium, and mercury. Main contamination sources: heavy metals in food appear mainly due to air, water and soil contamination.
	Natural toxins	Include mycotoxins, marine biotoxins, cyanogenic glycosides, and toxins in poisonous mushrooms. Symptoms: long-term exposure to these toxins can affect the immune system and normal development, or cause cancer. Main contamination sources: staple foods such as corn or cereals can contain high levels of mycotoxins, produced by mold present in the grain.
	Persistent organic pollutants	Compounds that accumulate in the environment and the human body. The best-known examples are dioxins and polychlorinated biphenyls, unwanted by products of industrial processes, and waste incineration. Symptoms: dioxins can cause reproductive and developmental problems, damage the immune system, interfere with hormone function, and cause cancer. Main contamination sources: these substances accumulate in the environment and the animal kingdom, which results in its intake by humans.
Prions		Infectious agents composed of proteins associated with certain types of neurodegenerative diseases. Bovine spongiform encephalopathy (or “mad cow disease”) is an example of prion disease that affects cattle associated with the variant Creutzfeldt-Jakob disease in humans. Consumption of meat products from cattle that contain specified risk materials, such as brain tissue, is the most likely route of transmission of the prion to humans.

Note: Own elaboration based on the WHO, [41].

**Table 2:** Requirements of the Ecuadorian law that regulate GMP in restaurants.

Requirements	Item Summary
Areas	Are properly defined and adequately maintained. The flammable products storage area complies with the provisions: away from the establishment, ventilated.
Basic services	Connected to the electrical network. Adequate drinking water supply and distribution system.
Behavior of staff	Is strictly regulated. Smoking and eating are not allowed in work areas. The hair must be completely covered to avoid physical contamination. The staff have clean, short nails. Staff must cover beards/mustaches. The use of jewelry and makeup should be avoided.
Ceilings	They are made of resistant, smooth, waterproof, easy-to-clean materials. They are in good and perfect cleaning conditions and do not allow dust accumulation.
Cleaning and disinfection	Are carried out daily. Cleaning and disinfection procedures include at least a combination of physical and chemical methods for cleaning surfaces.
Design and Building	Protects internal areas from: dust, rodents, birds, other polluting elements. The type of construction is solid.
Drains	Comply with the following provisions: adequate protection, easy maintenance, and cleaning. Drains have hydraulic seals, grease traps and/or solids installed.
Education	Is carried out through a training program on GMP. The requirements that the personnel must meet for each work area are defined. Restaurants have staff evaluation programs.
Equipment and other accessories	Have appropriate height. There is enough space to allow cleaning. The equipment and utensils are exclusive for each area. The equipment is made of resistant and non-toxic materials.
Floors	Are made of resistant, smooth, waterproof, easy-to-clean materials. They are kept in good and clean condition.
Health condition	Assures that the staff has a valid health card. It has preventive medicine programs. A registry of infectious diseases or skin lesions is kept. Personnel with infectious diseases or injuries are isolated.
Hygiene and protection	Follows standards of cleanliness and hygiene for staff. Provides the establishment of appropriate white clothing/uniforms for daily tasks. Uniforms are washable or disposable.
Hygienic service areas	Are separated by sex. Equipped with the necessary facilities. The floors, walls and windows are in a perfect state of cleanliness and good condition, organized and with adequate ventilation.
Illumination	Is adequate. Lighting does not alter the color of the products. There is protection on the lamps to prevent glass pieces from falling on the food in case of breakage.
Location	Is free from contamination and sources of unsanitary conditions.
Marketing conditions	Are adequate for each type of food. Showcases, shelves or furniture are easy to clean. The owner is responsible for the sanitary conditions that the food requires for its conservation.
Pest and pesticide control	Are kept closed and identified. Food scraps are cleaned at the end of each day and floors are swept daily. Garbage is not deposited in proximity of the establishment and is kept closed and identified.
Quality assurance and control	Are tailored to the needs of the process. Cleaning methods have procedures for the handling and application of chemical substances, that are used in cleaning and disinfection processes.
Reception of raw material	Is planned. The organoleptic characteristics of each type of product are verified. Arrival and storage temperatures are verified. Containers and cans are in good condition.
Solid waste disposal	Is an adequate system for collecting, depositing, and disposing of solid waste. Waste is collected in covered containers and identified. Waste is frequently removed from production areas.
Storage conditions	They are adequate and hygienic. Environmental conditions maintain the required temperatures and adequate shelves. Pest control programs are applied.
Walls	Wall surfaces should be made of smooth, washable, non-toxic, corrosion-resistant material, and maintained in a good condition.
Water	Transforms into ice with purified or treated water according to INEN regulations. There are records of physical, chemical, and microbiological controls of the water. The distribution system is identified.
Windows, doors, etc.	Are made of resistant, smooth, waterproof, easy-to-clean materials that do not fall off. They are in perfect clean conditions. The windows have glass and/or mosquito nets or similar.

**Table 3:** Leading causes of foodborne illness worldwide.

Food Group	Result of Microbiological Analysis			
	Total coliforms	*S. aureus*	*E. coli*	*Salmonella* spp.
Cooked fish	7.0 × 10^1^	<10	<3	Absence
Requirement	m:10 CFU/g	m:10 CFU/g	m < 3 MPN/g	m: Absence
Fresh juice	1.2 × 10^3^	<1.0	<1.0	Absence
Requirement	m:10 CFU/mL	m: * CFU/g	m:0 MPN/mL	m: Absence
Rice	3.0 × 10^3^	<10	<3	<3
Requirement	m:10 CFU/g	m:10 CFU/g	m < 3 MPN/g	m < 3 MPN/g
Salad	<10	<10	<10	Absence
Requirement	m:10^2^ CFU/g	m:10 CFU/g	m:10 CFU/g	m: Absence

Note: The values presented correspond to the average results in duplicate of samples composed of each food group. The requirements are taken from the NTC standards 5468:2007-02-21 JUICES (JUICES), NECTARS, PURES (PULPS) AND FROZEN OR UNFROZEN NON-PASTEURIZED FRUIT CONCENTRATES (for juices), NTS N—MINSA/DIGESA-V.01. XV.2. Prepared food heat treatment (for rice and cooked fish) and NTS N—MINSA/DIGESA-V.01. XV.1. Prepared food without heat treatment (for salads). Letter m indicates maximum permissible index to identify good quality, according to requirements of the standards. In this case, N, n, and c criteria were not used because samples were not from a lot. * The reference standard does not include a specific requirement for *S. aureus* in juices.

## Data Availability

Not applicable.
